# The interactive effects of emotional leadership and resilience on job satisfaction and organizational commitment among Korean youth sports instructors

**DOI:** 10.3389/fpsyg.2025.1534812

**Published:** 2025-04-16

**Authors:** Myung Kyu Jung, Tae Gyeom Jung, Yu Jin Kang, Kwon-Hyuk Jeong

**Affiliations:** ^1^Department of Taekwondo, College of Social Science & Culture, Dongshin University, Naju-si, Jeollanam-do, Republic of Korea; ^2^Taekwondo and Physical Education College, Shinhan University, Uijeongbu-si, Gyeonggi-do, Republic of Korea; ^3^Department of Physical Education, Seoul National University, Seoul, Republic of Korea; ^4^Department of Taekwondo, College of Physical Education, Kyung Hee University, Yongin-si, Gyeonggi-do, Republic of Korea

**Keywords:** emotional leadership, resilience, job satisfaction, organizational commitment, interactive effects

## Abstract

**Introduction:**

This study aims to provide practical insights into developing intervention strategies to enhance organizational effectiveness in Korean youth physical education facilities. Specifically, it investigates the interactive effects of resilience and emotional leadership on job satisfaction and organizational commitment.

**Methods:**

To address this aim, data were collected from 205 physical education instructors employed at Korean youth physical education facilities in 2024. The data were analyzed using Partial Least Squares Structural Equation Modeling (PLS-SEM).

**Results:**

The analysis revealed a statistically significant interaction effect between resilience and the personal competency dimension of emotional leadership on job satisfaction.

**Discussion:**

These findings provide a theoretical foundation for understanding the dynamic interaction between emotional leadership and resilience. Additionally, the results offer practical implications for developing effective human resource management strategies tailored to the unique organizational environment of Korean youth physical education facilities.

## 1 Introduction

Traditionally, job satisfaction and organizational commitment have been among the most representative research topics concerning organizational members. Job satisfaction refers to the positive emotions employees derive from performing their duties (Jeon et al., 2023; Jung et al., 2024; Locke, 1973, 1976), and organizational commitment refers to the level of attachment individuals feel toward their organization (Jung et al., 2024; Lee et al., 2023; Meyer et al., 1993; Williams and Anderson, 1991). Based on the premise that high levels of job satisfaction and organizational commitment not only improve organizational performance but also mitigate negative emotions, such as turnover intention (Folorunso et al., 2014; Jung et al., 2024; Maertz and Campion, 2004; Memari et al., 2013; Stinglhamber and Vandenberghe, 2003; Williams and Hazer, 1986; Wyland et al., 2016), the importance of both of these aspects has been increasingly emphasized in the context of enhancing organizational effectiveness.

The role and significance of job satisfaction and organizational commitment, rooted in their relationship with organizational effectiveness, have recently extended to youth sports instructors in South Korea. This is because, because of the characteristics of youth sports education facilities, where the interaction between service providers and recipients is crucial, human resources—specifically sports instructors—serve as the most critical factor in facility operation (Jeon et al., 2023; Morrell et al., 2004; Seo, 2018).

In particular, youth sports instructors in South Korea are exposed to various work environments beyond education, such as transportation duties, admission consultations, and facility promotion (Kang and Jung, 2023; Lee et al., 2023; Lee, 2017). Within the unique environmental characteristics of youth sports education facilities, where not only education but also caregiving roles are emphasized, the selfishness of some parents—who prioritize their own children and exhibit unreasonable demands—further exacerbates the negative emotions experienced by instructors during their work. Consequently, there is a growing demand for intervention strategies that can enhance the job satisfaction and organizational commitment of these instructors.

In this context, previous studies have highlighted resilience as a positive factor that enhances the organizational effectiveness of individual members when they face adversity and challenges (Yang and Chah, 2021; Youssef and Luthans, 2007). Resilience, in this sense, refers to the ability to overcome difficulties, adapt to the environment, and grow mentally (Connor, 2006; Garmezy, 1993; Luthar et al., 2000; Shin et al., 2009; Werner and Smith, 1982; Yang and Chah, 2021). It represents a multifaceted psychological capability that is composed of various elements, broadly categorized into three key components: control, which refers to the ability to recognize and regulate one’s emotions in difficult situations; sociality, which involves quickly understanding and empathizing with others’ emotional states; and positivity, which relates to enhancing control and sociality (Shin et al., 2009).

These resilience abilities—control, sociality, and positivity—are considered essential skills for youth sports instructors in South Korea. More resilient individuals are less likely to give up easily and more capable of overcoming difficulties through flexible responses, especially in high-stress job situations (Connor, 2006; Luthar and Cicchetti, 2000). This highlights the role and significance of resilience as a psychological intervention strategy fostering positive emotions, such as job satisfaction and organizational commitment. The effectiveness of resilience in influencing job satisfaction and organizational commitment among youth sports instructors in South Korean educational facilities is supported by empirical studies (Avcı Taşkıran et al., 2024; Brown et al., 2018; Youssef and Luthans, 2007). For example, Choi and Jung (2020) demonstrated the significant impact of resilience on the job satisfaction and organizational commitment of instructors working in taekwondo dojang, a representative youth sports education facility in South Korea. Similarly, Lee (2021) confirmed that resilience, particularly in terms of positivity, enhances job satisfaction and organizational commitment among instructors in hapkido dojang, another youth sports education facility in Korea.

Most youth sports education facilities in South Korea generally operate on a relatively small scale, with about 10 members. Therefore, the managers of these facilities are expected to play a significant role as both supervisors and leaders of the instructors in the relationship between resilience, job satisfaction, and organizational commitment. In particular, the small-scale nature of these facilities is likely to amplify the effectiveness of emotional leadership, as managers are directly engaged in various operational tasks alongside sports instructors. These tasks extend beyond the provision of sports education and include responsibilities such as admission consultations, transportation services, and other administrative duties. Therefore, it is anticipated that emotional leadership may interact with resilience and serve as a moderating factor in enhancing the effectiveness of resilience on the job satisfaction and organizational commitment of sports instructors.

Emotional leadership refers to an effective organizational management approach that, based on emotional intelligence (the ability to assess and regulate one’s own and others’ emotions, and to utilize them to plan, achieve, and manage one’s life) focuses on understanding, considering, and managing the emotional aspects of organizational members in order to exert a positive influence toward achieving organizational goals and visions (Goleman, 1995; Kwon and Sim, 2024; Salovey and Mayer, 1990). This type of leadership manifests through personal competencies, such as self-awareness (the ability to understand one’s emotions and how they affect others) and self-management (the ability to control and alter negative emotions). It also includes social competencies, such as social awareness (the ability to understand others’ emotions and respond accordingly) and relationship management (the ability to form and manage interpersonal relationships; Goleman et al., 2002; Kang and Jung, 2023; Salovey and Mayer, 1990; Sung et al., 2019).

Barsade and Gibson (2007) emphasized that leaders’ emotions are closely related to the psychology of their subordinates. Other studies, practically investigating theoretical premises on the potential of moderation effects, suggest that the efficacy of resilience and emotional leadership on job satisfaction and organizational commitment can be empirically examined among Korean youth sports instructors. They have theoretically posited the potential moderating effects of emotional leadership.

Despite these criticisms, previous studies on the relationship between resilience, job satisfaction, and organizational commitment have not seriously considered the role of emotional leadership. In particular, despite the distinct characteristics of youth sports facilities in South Korea, there is a significant lack of previous research conducted on these variables among sports instructors in the country. The few related studies have primarily focused on verifying the effects of resilience and emotional leadership on job satisfaction and organizational commitment in a fragmented manner. As a result, the moderating effect of emotional leadership on the relationship between resilience, job satisfaction, and organizational commitment—specifically, the interaction effect between resilience and emotional leadership on job satisfaction and organizational commitment—has not been adequately examined. A confirmed effect of the independent and moderating variables on the dependent variable indicates a corresponding effect of the moderating variable on the relationship between the independent and dependent variable.

Therefore, by focusing on the interaction effect between resilience and emotional leadership, this study aims to explore the in-depth relationships among the resilience, job satisfaction, and organizational commitment of youth sports instructors in South Korea, and the emotional leadership perceived by them. This approach will be significantly different from existing research models by way of providing a more comprehensive understanding of the relationships between these variables. Furthermore, it is expected to offer practical and valuable information for enhancing and fostering organizational effectiveness within youth sports education facilities in South Korea.

## 2 Research hypotheses

Given the unique environmental characteristics of youth sports education facilities in South Korea, the levels of job satisfaction and organizational commitment among current sports instructors have been declining. Resilience has emerged as a key predictor of these outcomes. Numerous studies (Avcı Taşkıran et al., 2024; Brown et al., 2018; Youssef and Luthans, 2007) have pointed out that individual job satisfaction and organizational commitment can vary according to the level of resilience. For example, Choi and Jung (2020) and Lee (2021) confirmed that resilience could serve as a significant predictor of job satisfaction and organizational commitment among sports instructors in youth sports facilities, such as taekwondo and hapkido dojangs in South Korea. Other studies conducted on teachers and childcare workers, who have job characteristics similar to those of Korean youth sports instructors (Chae and Kim, 2019; Kang and Ahn, 2015; Kwak and Baek, 2020; Park and Lee, 2018), have also demonstrated the effectiveness of resilience in predicting job satisfaction and organizational commitment.

Amidst this, it is anticipated that emotional leadership, based on its characteristic role, will play a moderating role in the relationship between resilience and job satisfaction, as well as in organizational commitment. In other words, the interaction of resilience and emotional leadership may influence job satisfaction and organizational commitment. This expectation is supported by Barsade and Gibson (2007), who have suggested that the emotions of leaders are closely related to the psychological state of their subordinates, as well as by prior studies that have explored the possibility of a moderating effect within the context of interaction effects among Korean youth sports instructors (Baek et al., 2011; Choi and Jung, 2020; Lee et al., 2023; Lee, 2021).

Based on the above research trends, the following hypotheses were formulated to empirically verify the expected relationships among emotional leadership, resilience, job satisfaction, and organizational commitment in the context of youth sports instructors:

*H1-1*. Resilience and emotional leadership, based on personal competence, will have an interaction effect on job satisfaction.

*H1-2*. Resilience and emotional leadership, based on social competence, will have an interaction effect on job satisfaction.

*H2-1*. Resilience and emotional leadership, based on personal competence, will have an interaction effect on organizational commitment.

*H2-2*. Resilience and emotional leadership, based on social competence, will have an interaction effect on organizational commitment.

## 3 Methodology

### 3.1 Sample and procedure

To achieve the objectives of this study, we used snowball sampling (since a non-probability sampling method was used, there is a possibility of potential bias in the sample selection process), a non-probability sampling method, to collect data from sports instructors currently employed at youth sports education facilities in South Korea (as of 2024). Data were gathered through Google Forms (an online survey platform). To mitigate potential limitations of online surveys, trap questions (e.g., “Please select ‘7, strongly agree’ for this item.”) were included. After data collection, responses that did not follow the trap question instructions or exhibited specific response patterns (e.g., 2222222) were scrutinized; finally, 205 valid responses were selected for the final analysis. Ethics approval was granted by the Public Institutional Bioethics Committee designated by the MOHW (Republic of Korea) (approval number: PO1-202403-01-026). Informed consent was obtained from all the participants as part of the Google Forms (an online survey platform).

Regarding the demographic characteristics of the final sample, 136 respondents were male (66.3%) and 69 were female (33.7%). In terms of age distribution, 113 respondents were in their 20s (55.1%) and 92 were in their 30s (44.9%).

### 3.2 Measurement

In this study, surveys, which have been used widely in multiple studies, were employed as research instruments to measure the resilience, job satisfaction, and organizational commitment of youth sports instructors in South Korea and the emotional leadership perceived by them. Detailed information regarding the sources and composition of each survey is as follows.

First, the independent variable of resilience was measured using a scale developed by Shin et al. (2009), which was adapted to the Korean context based on related prior research (Diener et al., 1985; Duran, 1983; Reivich and Shatte, 2002; Seligman, 2002; Wiemann, 1977). The items used in studies on Korean youth sports instructors (Choi and Jung, 2020; Kim and Jung, 2021) were modified and refined through content validity verification and variable refinement processes, and the scale was restructured into three factors: control, sociability, and positivity.

Next, the moderating variable of emotional leadership was measured using a questionnaire based on Park et al. (2009) and Yoo and Jung (2024); this was further adapted to the Korean context and modified, based on the items used by Goleman et al. (2002). The modified instrument was restructured into four factors—self-awareness (3 items), self-management (6 items), social awareness (3 items), and relationship management (6 items)—through content validity verification and variable refinement processes.

Lastly, the dependent variables, namely job satisfaction and organizational commitment, were measured using scales originally developed by Williams and Anderson (1991) and Mowday et al. (1982), respectively. These scales were modified and refined through content validity verification and variable refinement processes to suit the context of the employees of youth sports facilities in South Korea. All items used a 7-point Likert scale, ranging from 1, “Strongly Disagree,” to 7, “Strongly Agree.” The survey measurements were first verified by three professors with a PhD in sports psychology for facial validity and content validity (i.e., appropriateness for wording and context).

### 3.3 Data analysis

To achieve the research objectives, we employed partial least squares structural equation modeling (PLS-SEM), using Smart PLS version 4.105. PLS-SEM is a variance-based SEM approach that differs from covariance-based SEM. It is particularly suitable for analyzing small sample sizes or non-normally distributed data, as is the case in this research (Hair et al., 2017).

Regarding the specific analytical process, a hierarchical component model (HCM) with a reflective-formative type was applied in this study. The disjoint two-stage approach was used as the method for estimating the HCM. Additionally, 5,000 bootstrapping iterations were performed to derive the results.

## 4 Results

### 4.1 Measurement model

In the process of applying the HCM in PLS-SEM, it is essential to ensure the appropriateness of both, the lower-order components (LOCs) measurement model and the higher-order component (HOC) measurement model (Becker et al., 2012; Shin, 2004). The results of the analysis are presented separately for the LOC and HOC measurement models as follows.

For the reflective measurement model of the first-order LOCs, the analysis results showed factor loadings ranging from 0.902 to 0.980 (>0.70), average variance extracted (AVE) ranging from 0.881 to 0.949 (>0.50), Cronbach’s alpha ranging from 0.974 to 0.988 (>0.70), composite reliability (CR) ranging from 0.981 to 0.990 (>0.70), and rho_a ranging from 0.974 to 0.989 (>0.70). These results, shown in Table 1, suggest that convergent validity and internal consistency reliability were established on the basis of commonly accepted validation criteria. Furthermore, based on the Fornell–Larcker criterion, the discriminant validity between the first components was assessed as shown in Table 2. The square root of AVE was found to be greater than the maximum correlation coefficient between latent variables, suggesting that discriminant validity was also established.

**Table 1:** Analysis results of the first-order component measurement model.

Construct and Item		Convergent validity		Internal consistency reliability		
		Outer loading (L)	AVE	*a*	CR	rho_a
Control of resilience (CoR)	CoR 1	0.949	0.913	0.981	0.984	0.981
	CoR 2	0.956	0.913	0.981	0.984	0.981
	CoR 3	0.962	0.913	0.981	0.984	0.981
	CoR 4	0.956	0.913	0.981	0.984	0.981
	CoR 5	0.960	0.913	0.981	0.984	0.981
	CoR 6	0.951	0.913	0.981	0.984	0.981
Sociality of resilience (SR)	SR 1	0.951	0.911	0.980	0.984	0.981
	SR 2	0.950	0.911	0.980	0.984	0.981
	SR 3	0.954	0.911	0.980	0.984	0.981
	SR 4	0.965	0.911	0.980	0.984	0.981
	SR 5	0.957	0.911	0.980	0.984	0.981
	SR 6	0.950	0.911	0.980	0.984	0.981
Positivity of resilience (PR)	PR 1	0.965	0.945	0.988	0.990	0.989
	PR 2	0.970	0.945	0.988	0.990	0.989
	PR 3	0.974	0.945	0.988	0.990	0.989
	PR 4	0.974	0.945	0.988	0.990	0.989
	PR 5	0.979	0.945	0.988	0.990	0.989
	PR 6	0.970	0.945	0.988	0.990	0.989
Personal competence emotional leadership (PCEL)	PCEL 1	0.909	0.915	0.984	0.987	0.985
	PCEL 2	0.963	0.915	0.984	0.987	0.985
	PCEL 3	0.969	0.915	0.984	0.987	0.985
	PCEL 4	0.968	0.915	0.984	0.987	0.985
	PCEL 5	0.954	0.915	0.984	0.987	0.985
	PCEL 6	0.970	0.915	0.984	0.987	0.985
	PCEL 7	0.962	0.915	0.984	0.987	0.985
Social competence emotional leadership (SCEL)	SCEL 1	0.902	0.881	0.977	0.981	0.978
	SCEL 2	0.939	0.881	0.977	0.981	0.978
	SCEL 3	0.946	0.881	0.977	0.981	0.978
	SCEL 4	0.958	0.881	0.977	0.981	0.978
	SCEL 5	0.939	0.881	0.977	0.981	0.978
	SCEL 6	0.951	0.881	0.977	0.981	0.978
	SCEL 7	0.933	0.881	0.977	0.981	0.978
Job satisfaction (JS)	JS 1	0.969	0.949	0.982	0.987	0.982
	JS 2	0.971	0.949	0.982	0.987	0.982
	JS 3	0.980	0.949	0.982	0.987	0.982
	JS 4	0.978	0.949	0.982	0.987	0.982
Organizational commitment (OC)	OC 1	0.959	0.927	0.974	0.981	0.974
	OC 2	0.968	0.927	0.974	0.981	0.974
	OC 3	0.967	0.927	0.974	0.981	0.974
	OC 4	0.956	0.927	0.974	0.981	0.974

AVE, average variance extracted; CR, construct reliability.

**Table 2:** Results of discriminant validity testing among first-order components based on the Fornell–Larcker criterion.

	A. CoR	B. SR	C. PR	D. PCEL	E. SCEL	F. JS	G. OC
A	***0.956***						
B	0.745	***0.954***					
C	0.700	0.763	***0.972***				
D	0.761	0.791	0.728	***0.957***			
E	0.732	0.729	0.675	0.729	***0.939***		
F	0.765	0.741	0.724	0.783	0.766	***0.974***	
G	0.745	0.795	0.686	0.725	0.730	0.769	***0.963***

Values in bold, italicized = square root of the AVE (Average variance extracted) of that factor.

CoR, control of resilience; SR, sociality of resilience; PR, positivity of resilience; PCEL, personal competence emotional leadership; SCEL, social competence emotional leadership; JS, job satisfaction; OC, organizational commitment.

For the formative measurement model of the second-order components (HOC), the analysis results confirmed their appropriateness by verifying the significance of the outer weights of the first-order components, as well as confirming outer loadings above 0.50 and their significance. Additionally, variance inflation factors values were detected to be below 5 for all first-order components, indicating no multicollinearity issues among them (Table 3).

**Table 3:** Analysis results of the formative measurement model for the second-order component.

HOC	LOCs	Outer weights (W)	*t*	*p*	Outer loading (L)	*t*	*p*	VIF
Resilience	Control	0.438	5.430***	<0.001	0.918	36.240***	<0.001	2.476
	Sociality	0.451	5.294***	<0.001	0.934	37.796***	<0.001	3.022
	Positivity	0.206	3.191**	<0.01	0.857	24.539***	<0.001	2.641

HOC, higher-order component; LOCs, lower-order components. ***p < 0.001, **p < 0.01.

### 4.2 Structural model

In this study, cross-validation (Q^2^), based on blindfolding, was conducted to assess the predictive relevance of the structural model. The results showed that the Q^2^ values for job satisfaction and organizational commitment were 0.681 and 0.713, respectively, which are above 0, indicating adequate predictive relevance (Hair et al., 2017). Based on this relevance, the interaction effects between resilience and emotional leadership on job satisfaction and organizational commitment were examined, and the results are as follows.

**Figure 1:**
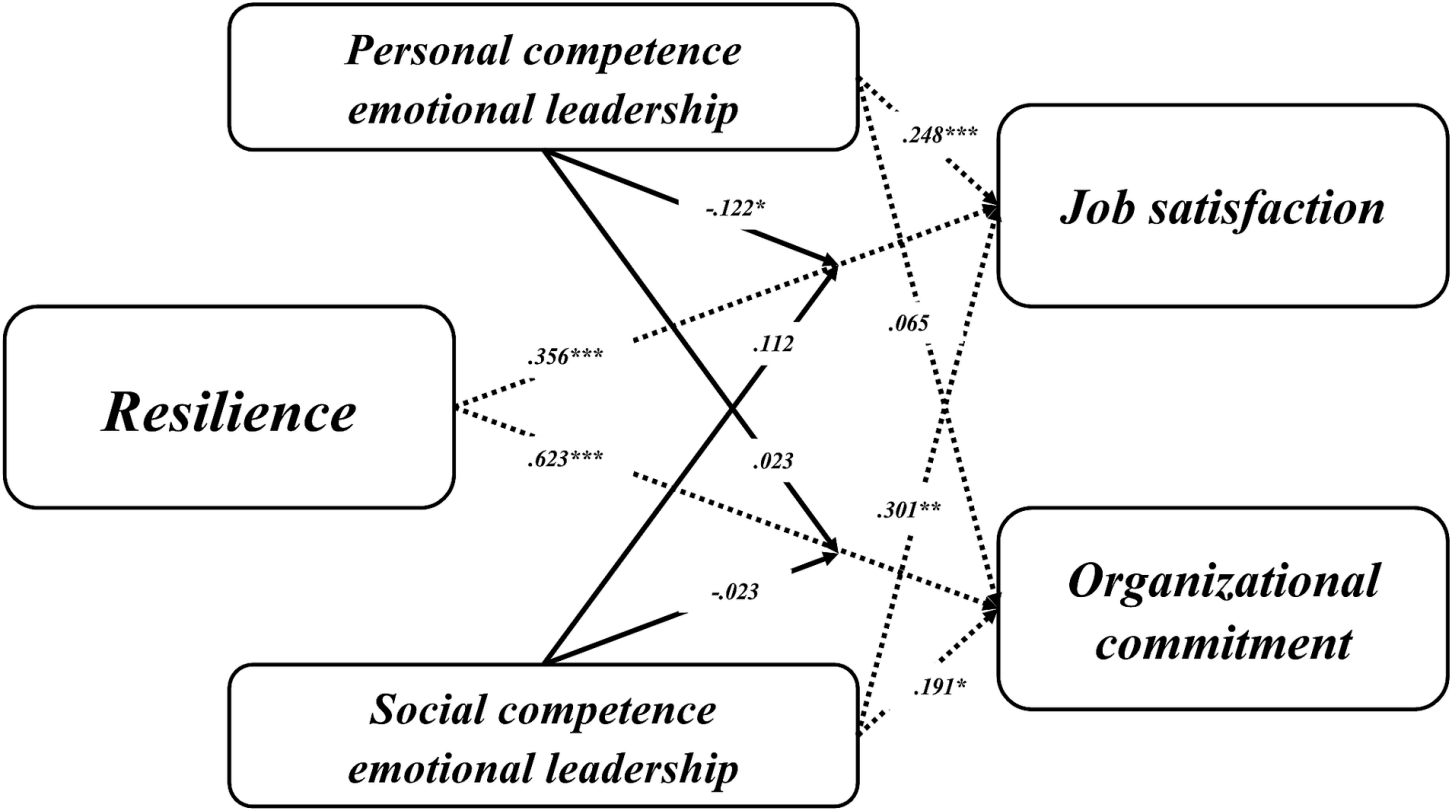
Research hypothesis testing models.

As shown in Table 4 and Figure 1, while the significance of all direct causal relationships was examined, except for that between emotional self-awareness and organizational commitment, the interaction effect was confirmed only in the context of job satisfaction, specifically between resilience and personal competency emotional leadership (H1-1). However, the other hypotheses, namely H1-2, H2-1, and H2-2, were rejected.

**Table 4:** Hypothesis testing results based on the two-stage approach.

Path	*b*	SE	*t*	*P*
Resilience → Job satisfaction	0.356	0.101	3.515	0.000
Resilience → Organizational commitment	0.623	0.093	6.701	0.000
Personal competence emotional leadership → Job satisfaction	0.248	0.066	3.751	0.000
Personal competence emotional leadership → Organizational commitment	0.065	0.073	0.893	0.372
Social competence emotional leadership → Job satisfaction	0.301	0.100	3.015	0.003
Social competence emotional leadership → Organizational commitment	0.191	0.074	2.589	0.010
Resilience × Personal competence emotional leadership → Job satisfaction	−0.122	0.055	2.225	0.026
Resilience × Personal competence emotional leadership → Organizational commitment	0.023	0.048	0.484	0.629
Resilience × Social competence emotional leadership → Job satisfaction	0.112	0.064	1.745	0.081
Resilience × Social competence emotional leadership → Organizational commitment	−0.023	0.052	0.449	0.654

## 5 Discussion

This study aims to provide valuable insights for developing effective intervention strategies to enhance organizational effectiveness in Korean youth sports education facilities. Specifically, it explores the relationships among resilience, emotional leadership, job satisfaction, and organizational commitment, with a focus on the interaction effects between resilience and emotional leadership. To achieve this objective, the hypotheses regarding the interaction effects of resilience and emotional leadership on job satisfaction and organizational commitment were tested.

The analysis results showed that, despite the effectiveness of resilience and emotional leadership on job satisfaction and organizational commitment among Korean youth sports instructors, only the interaction effect between resilience and personal aspects of emotional leadership on job satisfaction (H1) was observed to be significant. This finding suggests that the effectiveness of resilience on job satisfaction of youth sports instructors may vary by the level of emotional leadership pertaining to personal aspects, such as self-awareness and self-management. The observed findings are directly and indirectly supported by previous studies (Baek et al., 2011; Choi and Jung, 2020; Lee et al., 2023; Lee, 2021) that have discussed the theoretical premise of the potential moderating effects of interactions such as those observed in this study.

Leaders who effectively utilize emotional leadership are said to be capable of accurately recognizing and controlling their own emotions. These emotions, when accurately recognized and controlled through emotional leadership, are closely related to the psychological aspects of organizational members (Barsade and Gibson, 2007; Goleman et al., 2002; Yang and Chah, 2021). Emotional leadership contributes to higher employee satisfaction, improved organizational productivity, and the creation of positive organizational culture. Emotional leadership is especially important in today’s complex and diverse society in order to meet the varied needs of employees and drive innovation (Caruso et al., 2001). The observed results suggest that leaders with strong emotional leadership can foster a more positive emotional climate among organizational members. This ultimately results in the moderating effect of emotional leadership in personal aspects on the relationship between resilience and job satisfaction.

It is worth noting that the observed interaction effect between resilience and personal aspects of emotional leadership on job satisfaction demonstrated a moderating effect in a mitigating manner. This implies that, although emotional leadership in personal aspects plays a positive role, the interaction results suggest that the effect of resilience on job satisfaction may decrease at higher levels of personal competency in emotional leadership.

Managers who demonstrate emotional leadership in personal aspects tend to perform their duties according to their own subjective judgment (Goleman et al., 2002; Yang and Chah, 2021). However, given the unique characteristics of Korean youth sports education facilities—such as their relatively small operational scale and the fact that sports instructors often perform various tasks such as educational instruction, vehicle operation, consultation, and facility promotion alongside the managing leader (Kang and Jung, 2023; Lee et al., 2023; Lee, 2017)—emotional leadership in personal aspects could sometimes drive unilateral decision-making centered on the leader. This, as observed in the results, may reduce the effectiveness of resilience on job satisfaction, which is based on autonomy, because of the leader’s strong personal emotional leadership. As an important trait for leaders with good emotion management and an excellent predictor of leadership emergence, empathy enables both task- and relationship-oriented skills (Kellett et al., 2002). Choi (2019) has suggested that, for youth sports club managers to increase job satisfaction and organizational attachment to leaders through emotional leadership, they need to create an environment that is conducive to creating stronger connections and communication with leaders, wherein leaders can freely share opinions and express their feelings. Therefore, it is crucial to develop intervention strategies that help leaders guide their organizational members by exerting an appropriate level of emotional leadership in personal aspects.

In summary, when utilizing resilience as an effective intervention strategy to enhance organizational effectiveness in Korean youth sports education facilities, it is important to understand that excessive emotional leadership in personal aspects can negatively affect the relationship between resilience and job satisfaction. Leaders, particularly those in managerial positions, should be guided to exhibit an appropriate level of emotional leadership. Therefore, it is essential to engage in various discussions on personal aspects of emotional leadership, such as self-awareness and self-management, tailored to the characteristics of Korean youth sports instructors, and attempt to develop effective intervention strategies that can be practically applied within youth sports education facilities. For example, managers may seek to effectively exercise emotional leadership by clearly understanding how sports instructors perceive emotional leadership within youth sports education facilities through interviews, counseling, or other forms of communication.

## 6 Conclusion

This study aimed to provide practical insights for developing intervention strategies to improve organizational effectiveness in Korean youth sports education facilities by investigating the interaction effects of resilience and emotional leadership on job satisfaction and organizational commitment. The findings revealed that the emotional leadership of managers and the resilience of youth sports instructors interact to influence the instructors’ level of job satisfaction. Based on these results, the study underscores the importance of identifying the appropriate level of emotional leadership for Korean youth sports instructors and developing effective intervention strategies that can be practically applied within these facilities.

Given the limited prior research on the interaction effects of resilience and emotional leadership on job satisfaction and organizational commitment among youth sports instructors, this study serves as a foundational step toward enhancing organizational effectiveness in the context of Korean youth sports education. The academic and practical implications derived from this research are meaningful; however, further studies are required to accumulate more objective and consistent evidence supporting these findings. Accordingly, this study concludes with the following suggestions, grounded in its limitations.

First, as this research constitutes the first direct attempt to verify the interaction effects of resilience and emotional leadership on job satisfaction and organizational commitment among Korean youth sports instructors, future studies should incorporate additional variables closely related to these factors. Such efforts would help to provide a more comprehensive understanding of the complex relationships among emotional leadership, resilience, job satisfaction, and organizational commitment.

Second, in light of the clear limitations in discussing the rejected moderating effects—largely due to the absence of direct prior research—follow-up studies are needed to validate the findings. These studies should consider potential factors such as the limitations of the sampling method adopted in this study and possible conceptual overlaps among the variables.

Third, the current study has inherent limitations related to its cross-sectional design and the inability to fully control for external variables that may influence the psychological factors of the participants (e.g., salary levels, specific industry sectors, job characteristics). Therefore, future research should adopt a more multidimensional approach to provide broader and deeper insights into the relationships between emotional leadership, resilience, job satisfaction, and organizational commitment.

Fourth, as this study was conducted within the specific context of youth sports education facilities in South Korea, the generalizability of its findings is limited. Thus, it is recommended that future studies be conducted in youth education and sports settings in other countries with similar organizational characteristics to examine whether the findings and implications of this study can be extended to international contexts.

## Data Availability

The original contributions presented in the study are included in the article/supplementary material, further inquiries can be directed to the corresponding author.
